# Catalytic Ozonation of Pharmaceuticals Using CeO_2_-CeTiO_x_-Doped Crossflow Ultrafiltration Ceramic Membranes

**DOI:** 10.3390/nano14131163

**Published:** 2024-07-07

**Authors:** Nikoletta Tsiarta, Silvia Morović, Vilko Mandić, Ivana Panžić, Roko Blažic, Lidija Ćurković, Wolfgang Gernjak

**Affiliations:** 1 Catalan Institute of Water Research, Carrer Emili Grahit 101, 17003 Girona, Spain; ntsiarta@icra.cat; 2 Campus de Montilivi, University of Girona, 17003 Girona, Spain; 3 Faculty of Mechanical Engineering and Naval Architecture, University of Zagreb, Ivana Lučića 5, 10002 Zagreb, Croatia; lidija.curkovic@fsb.unizg.hr; 4 Faculty of Chemical Engineering and Technology, University of Zagreb, 10000 Zagreb, Croatia; smorovic@fkit.unizg.hr (S.M.); vmandic@fkit.unizg.hr (V.M.); ipanzic@fkit.unizg.hr (I.P.); rblazic@fkit.unizg.hr (R.B.); 5 Catalan Institution for Research and Advanced Studies (ICREA), 08010 Barcelona, Spain

**Keywords:** crossflow filtration, ceramic membrane, catalytic ozonation, hybrid process, hydroxyl radicals

## Abstract

The removal of persistent organic micropollutants (OMPs) from secondary effluent in wastewater treatment plants is critical for meeting water reuse standards. Traditional treatment methods often fail to adequately degrade these contaminants. This study explored the efficacy of a hybrid ozonation membrane filtration (HOMF) process using CeO_2_ and CeTiO_x_-doped ceramic crossflow ultrafiltration ceramic membranes for the degradation of OMPs. Hollow ceramic membranes (CM) with a 300 kDa molecular weight cut-off (MWCO) were modified to serve as substrates for catalytic nanosized metal oxides in a crossflow and inside-out operational configuration. Three types of depositions were tested: a single layer of CeO_2_, a single layer of CeTiO_x_, and a combined layer of CeO_2_ + CeTiO_x_. These catalytic nanoparticles were distributed uniformly using a solution-based method supported by vacuum infiltration to ensure high-throughput deposition. The results demonstrated successful infiltration of the metal oxides, although the yield permeability and transmembrane flow varied, following this order: pristine > CeTiO_x_ > CeO_2_ > CeO_2_ + CeTiO_x_. Four OMPs were examined: two easily degraded by ozone (carbamazepine and diclofenac) and two recalcitrant (ibuprofen and pCBA). The highest OMP degradation was observed in demineralized water, particularly with the CeO_2_ + CeTiO_x_ modification, suggesting O_3_ decomposition to hydroxyl radicals. The increased resistance in the modified membranes contributed to the adsorption phenomena. The degradation efficiency decreased in secondary effluent due to competition with the organic and inorganic load, highlighting the challenges in complex water matrices.

## 1. Introduction

Urbanization and industrialization have led to the generation of vast quantities of wastewater containing diverse organic micropollutants (OMPs), including pharmaceuticals and personal care products, which pose significant challenges to traditional treatment methods [1,2]. The need for effective wastewater treatment is further underscored by the growing water scarcity intensified by climate change and erratic rainfall patterns. In response to these challenges, the concept of water reuse (also known as water recycling or water reclamation) has gained prominence as a sustainable solution to augment declining freshwater resources [3]. By treating wastewater to a high standard, it can be safely reused for various non-potable applications, such as irrigation, industrial processes, and environmental restoration.

Water reuse not only helps alleviate the strain on freshwater sources but also contributes to the circular economy by conserving resources and minimizing waste generation. In regions facing water scarcity, such as southern parts of Europe (Spain, Cyprus, Italy, and Greece), water reuse has become integral to ensuring water security and resilience in the face of climate variability (WFD 2000/60/EC [4], EU 2020/2184 [5]). Moreover, by diverting treated wastewater from discharge into water bodies, water reuse helps mitigate pollution and protects aquatic ecosystems from the adverse impacts of OMPs and other contaminants.

In light of these considerations, the European Union (EU) has implemented directives and regulations to promote water reuse and establish quality standards for recycled water [4,6]. These directives aim to facilitate the safe and sustainable reuse of treated wastewater for agricultural, industrial, and urban applications while safeguarding human health and the environment. By setting stringent criteria for water quality and treatment processes, the EU directives ensure that recycled water meets the required standards for its intended use, thereby promoting public acceptance and confidence in water reuse practices.

In response to these challenges, researchers have been exploring innovative treatment approaches to efficiently remove OMPs from wastewater. Hybrid treatment processes, which combine multiple technologies to synergistically target different classes of pollutants, have emerged as promising solutions [7,8]. Among these hybrid approaches, ozonation combined with membrane filtration has gained traction due to its versatility and effectiveness in removing a wide range of contaminants. Ozone (O_3_) is a powerful oxidizing agent that can react with organic compounds present in wastewater, leading to their degradation into simpler, less harmful by-products [9,10,11]. When coupled with membrane filtration, which physically separates suspended solids and microorganisms, ozonation offers a comprehensive treatment solution for complex wastewater streams [12,13].

While membrane filtration has been widely adopted in wastewater treatment plants, the choice of membrane material significantly influences the treatment efficiency and longevity [14,15,16]. Traditional polymeric membranes, although cost-effective, are prone to fouling, chemical degradation, and a limited lifespan under harsh operating conditions [17]. In contrast, ceramic membranes have garnered attention for their superior chemical and mechanical stability, resistance to fouling, and longer service life [18]. Composed of inorganic materials such as alumina, zirconia, or titania, ceramic membranes (CMs) exhibit enhanced durability and reliability in wastewater treatment applications, making them well-suited for challenging environments [19,20,21,22].

A number of recent studies have investigated heterogeneous catalytic ozonation coupled with membrane filtration in an attempt to achieve better removal of OMPs and their transformation products, supporting a reduction in toxicity after treatment [23,24,25,26]. So far, researchers have proposed the following modifications to enhance OMPs reduction: MnO_2_-Co_3_O_4_ [27], MnO_2_ [28], Ce-Ti composites [29], ZnO_2_ [30], CeO_x_ and MnO_x_ [31], and Co_3_O_4_-Al_2_O_3_ [32], supporting a reduction in toxicity after treatment. In all of them, hydroxyl radical (^•^OH) exposure was drastically increased; however, fouling was a problem. Therefore, efficient coupling of ozone to CM, adjusting the ozone dosage and the catalytic material used, is needed to prevent severe membrane fouling. In very recent studies (2024), He et al. [33] investigated the effect of Fe(II)/KMnO_4_ on the fouling of ceramic membranes and found that ozonation mitigates fouling caused by humic acid as it is decomposed into reactive radicals. Additionally, Liangby et al. [34] used Fe_3_O_4_-modified ceramic membranes during ozonation, and fouling by humic acids was also reduced during atrazine treatment. 

Different methods exist for applying thin film layers of metal oxides to other materials. Common methods include Physical Vapor Deposition (PVD), Chemical Vapor Deposition (CVD), Sol-Gel Processing, and Atomic Layer Deposition (ALD). PVD techniques like sputtering and evaporation transfer material from a source to a substrate in a vacuum, resulting in high-purity films with excellent adhesion and controlled thickness [35]. CVD involves the chemical reactions of gaseous precursors on a heated substrate, forming a solid film with excellent conformality and uniformity over complex geometries, making it ideal for high-quality films of various materials [36]. Sol-gel processing involves hydrolysis and condensation of metal alkoxides to form a colloidal suspension (sol) that transitions into a gel and then a thin film upon drying and heating, producing high homogeneity and purity films at a lower cost [37]. ALD is a sequential, self-limiting process that deposits films one atomic layer at a time. It provides precise control over the thickness and composition, offering excellent uniformity and conformality at the atomic scale, making it ideal for ultrathin films. For instance, Liu and He [38] applied ALD to create an ultra-thin layer of Al_2_O_3_ on a membrane with great potential for industrial H_2_ purification and recovery. 

In this study, a novel approach that integrates catalytic ozonation with crossflow ultrafiltration using tubular ceramic membranes doped with metal oxides with a vacuum infiltration technique is proposed. Specifically, the deposition of cerium oxide (CeO_2_), cerium-doped titanium oxide (CeTiO_x_), and their combination onto ceramic substrates to enhance the catalytic activity during ozonation [31,39] is investigated. The choice of these metal oxides is motivated by their known catalytic properties, which can potentially accelerate the degradation of pharmaceuticals and other recalcitrant micropollutants. Herein, the degradation of carbamazepine (CBZ), diclofenac (DCF), ibuprofen (IBP), and para-chlorobenzoic acid (pCBA) in the presence or absence of a scavenger, such as tertiary butanol (TBA), and in real secondary effluent from the Girona’s wastewater treatment plant (WWTP) is tested. 

The hypothesis guiding our research is that modifying ceramic membranes with CeO_2_, CeTiO_x_, or their combination will enhance the degradation of recalcitrant OMPs, such as IBP and pCBA, during ozonation. By leveraging the catalytic activity of these metal oxides, we aim to achieve improved pollutant removal efficiency and overall treatment performance. Through systematic experimentation and analysis, we seek to validate this hypothesis and contribute to the advancement of hybrid ozonation–membrane filtration systems for urban wastewater treatment.

## 2. Materials and Methods

### 2.1. Chemicals and Materials

In this study, carbamazepine (CBZ), ibuprofen (IBP), and diclofenac (DCF) were used as model pharmaceutical compounds because they are frequently detected in secondary effluents. CBZ (99.8%, C_15_H_12_N_2_O), IBP (98.9%, C_13_H_18_O_2_), and DCF sodium salt (98%, C_14_H_10_C_12_NNaO_2_) were purchased from Sigma-Aldrich (St. Louis, MO, USA). Para-chlorobenzoic acid (pCBA, 99%, C_7_H_5_ClO_2_) was used as an O_3_/^•^OH probe compound, and it was purchased from ACROS Organics (Waltham, MA, USA). The physicochemical characteristics of the model compounds are summarized in Table S1. Sodium bicarbonate (NaHCO_3_) was used as a buffer to avoid variations in the pH, and it was obtained from Sigma-Aldrich. For the filtration experiments, single-tubular commercial ceramic membranes (CM) with 10 mm outer diameter, 6 mm inner diameter, 250 mm length, and 300 kDa molecular weight cut-off (MWCO) were utilized (*INSIDE*CéRAM, TAMI Industries, Nyons, France). Table S2 summarizes the characteristics of the CMs. For the modification of the CMs, the following chemicals were used: titanium (IV) isopropoxide (TIP, 97%, C_12_H_28_O_4_Ti, Sigma-Aldrich), cerium (III) nitrate hexahydrate (ceria, 99.5%, CeN_3_O_9_·6H_2_O, ACROS Organics), citric acid (C_6_H_8_O_7_, Sigma-Aldrich), nitric acid (HNO_3_, Sigma Aldrich) isopropanol (IPA, C_3_H_8_O, Grammol, Zagreb, Croatia, HR), acetylacetone (AcAc, ≥99%, C_5_H_8_O_2_, Honeywell, Charlotte, NC, USA) and polyvinyl alcohol (PVA, (C_2_H_4_O)_x_). In all the ozonation experiments, thiosulfate (Na_2_S_2_O_3_, Sigma Aldrich) was used to quench the reaction. For the scavenging tests, tertiary butanol (TBA, C_4_H_10_O, Sigma Aldrich) was used as a scavenger of hydroxyl radicals (^•^OH). The mobile phase of the liquid chromatography was prepared with ultrapure water (Millipore, Darmstadt, Germany), acetonitrile (ACN, CH_3_CN, Merck, Darmstadt, Germany), and formic acid (FA, CH_2_O_2_, Sigma-Aldrich). 

### 2.2. Preparation of Modified Ceramic Membranes

The CMs were modified by the sol-gel method under vacuum infiltration conditions to allow good sol dispersion (colloidal solution) through the membrane’s pores and surface. Two different sols were prepared, ceria (CeO_2_) and Ce-doped Ti (CeTiO_x_) with 1% mol Ce with regards to Ti [39]. Three nanosized films were separately deposited on the surface of each CM: one layer of CeO_2_ [31], one layer of CeTiO_x_ (TiO_2_ [40] adjusted to [39]), and one layer of CeO_2_ with an additional layer of CeTiO_x_ (CeO_2_ + CeTiO_x_). The sol-gel-assisted wet infiltration method was used for the modification of the CMs. The ceria precursor solution consisted of 0.5 M cerium (III) nitrate and 1.0 M citric acid in ultrapure water and was prepared and stirred overnight to achieve colloidal properties. The titania precursor solution containing 1% mol Ce in regard to Ti was prepared by dissolving 1.41 g cerium (III) nitrate in 1:10:10 *v*/*v* 0.5 M HNO_3_:IPA:TIP solution by adding AcAc and IPA. The calculations were adjusted from Ćurković et al. [40]. A step-by-step procedure is described in the Supplementary Materials (Text S1). 

### 2.3. Characterization Techniques

The pristine ceramic membranes and their modified surfaces were characterized to determine their chemical and phase composition, structure, and morphology using the following analytical techniques: Scanning Electron Microscopy (SEM) with Energy Dispersive X-ray Spectroscopy (EDS), X-ray Diffraction Analysis (XRD), and Atomic Force Microscopy (AFM). Mercury intrusion porosimeter (MIP) was used to evaluate the porosity, pore size distribution, and pore volume. 

The morphology of the CMs was characterized using a Tescan VEGA 3 microscope (TESCAN GROUP, Brno, Czech Republic). To enhance the electrical conductivity, all the samples were sputter-coated with a gold/palladium alloy in an argon plasma for 120 s prior to analysis. SEM analysis was conducted at an accelerating voltage of 20 kV. Additionally, elemental analysis of the CMs was performed using an energy dispersive X-ray spectrometry (EDS) detector (Bruker) at an accelerating voltage of 20 kV and working distance of 20 mm.

XRD was performed using a Bruker D8 diffractometer (Bruker Corporation, Billerica, Massachusetts, USA) with CuKα radiation. Data were collected in a step scan mode with steps of 0.02° 2θ and a counting time of 0.2 s in the range of 10 to 75° 2θ. Samples were recorded as powders and in a Bragg–Brentano configuration.

AFM images were taken using Nanosurf CoreAFM, Singapore under ambient conditions. Non-contact (tapping) mode was used for the acquisition, with a setpoint of 35%, a Tap300Al-G tip with a nominal spring constant of 40 N m^−1^, a tip radius less than 10 nm, and a nominal resonant frequency of 300 kHz on a 10 × 10 μm surface, and a 0.78 s acquisition time. Images were processed with Nanosurf software (CoreAFM, v 3.10.5).

MIP analysis was performed with the AutoPore IV 9500 mercury porosimeter (Merimetrics, Norcross, GA, USA), covering the pore diameter range from approx. 360 to 0.005 μm. A high pressure of up to 400 MPa was used to measure the pore structure parameters for each membrane. The principle of porosimetry mercury measurements is that as mercury is forced into a material under increasing pressure, the relationship between the pressure and the pore diameter can be determined using the Washburn equation [41]. 

### 2.4. Permeability Tests

Permeability tests were performed to examine the changes after the deposition of the nanosized films. Demineralized (DI) water was used in all the tests, and the retentate flow, the permeate flow, the temperature, and the pressure before and after the membrane module were recorded. The transmembrane flow (or permeate water flow) was calculated using Equation (1) as set out in the manual for operating the SIVALAB crossflow filtration pilot unit.

(1) $$DE\;1\;25=\frac{J_{w}\times K_{t}}{TMP\times A}$$

where DE 1 25 is the permeate water flow (L m^−2^ h^−1^) for a transmembrane pressure of 1 bat at 25 °C, *J*_w_ is the measured permeate water flow (L h^−1^), *K*_t_ is the temperature coefficient of water to convert it at 25 °C, TMP is the transmembrane pressure as the average of the pressures before and after the membrane (bar), and *A* is the area of the ceramic membrane (m^2^). Additionally, the hydraulic retention time (HRT) was calculated by Equation (2).

(2) $$HRT=\frac{V_{m}\times \varepsilon_{m}}{Q}$$

where HRT is the hydraulic retention time (s), *V*_m_ is the pore volume of the membrane (m^3^), *ε*_m_ is the porosity of the membrane, and *Q* is the permeate flow rate (m^3^ s^−1^). Finally, the membrane resistance (*R*_m_) was estimated by the following equation:

(3) $$R_{m}=\frac{TMP}{\mu \times J_{w}}$$

where *R*_m_ is the membrane resistance (m^−1^), TMP is the transmembrane pressure (Pa), *μ* is the water viscosity (8.90 × 10^−4^ Pa s at 25 °C), and *J*_w_ is the water flux in permeate (m s^–1^). The HRT and *R*_m_ were determined using the method described by Lee et al. [39]. 

### 2.5. Hybrid Ozonation–Membrane Filtration (HOMF) Tests

Ceramic membrane ultrafiltration experiments were carried out using a custom-built crossflow filtration unit with an inside-out configuration (SIVA, Nyons, France), as shown in Figure S2. The single tubular membranes (TAMI Industries, France) were 250 mm long, with 6 mm inner and 10 mm outer diameters, made of titanium dioxide support and an active zirconia oxide layer. The membranes were additionally coated with a layer of CeO_2_, CeTiO_x_, and a combination of CeO_2_ and CeTiO_x_. Before each experiment, the CMs were chemically cleaned with a 0.5 M NaOH solution and demineralized (DI) water.

A concentrated aqueous ozone solution (O_3(aq)_) was produced using a high-output ozone generator (ANSEROS, COM-AD-04, Tübingen, Germany) and maintained at ~2 °C. The ozone stock concentration was approximately 45 mg L^−1^ with a continuous cold DI water supply. Filtration experiments included the following: (a) filtration alone and (b) in situ ozonation and filtration at room temperature and 1 bar transmembrane pressure. An aqueous ozone was consistently introduced before filtration, and its concentration was monitored. A specially designed needle with a reduced diameter of 1 mm was placed at the ozone inlet, ensuring effective mixing with the feed stream.

**Figure 1:**
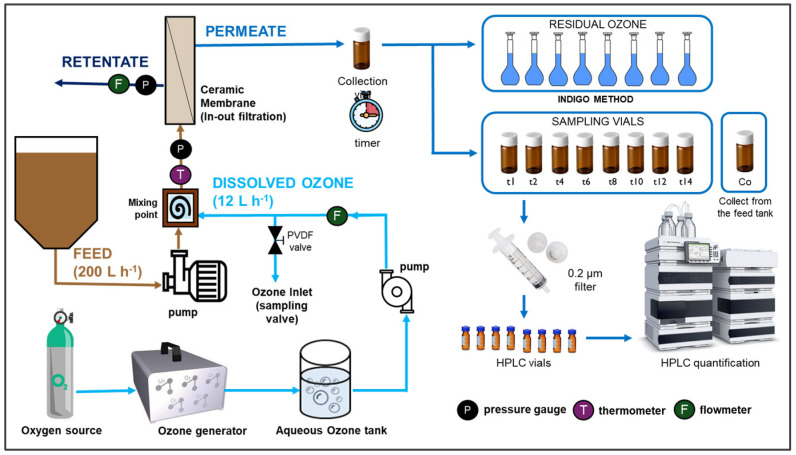
Schematic representation of the hybrid ozonation–membrane filtration (HOMF) process.

During the filtration experiments, the OMP-containing solution (feed) was mixed with DI water rather than an ozone solution. The membrane flux varied between the tested CMs. The experiments were performed with 10 μM OMP mix solution for 14 min. Permeate samples were collected every two minutes to determine the residual ozone and OMP concentrations. Feed and retentate samples were also collected and quenched with Na_2_S_2_O_3_ [42], then filtered through 0.2 μM nylon filters (Whatman, Maidstone, UK) before analysis (Figure 1).

### 2.6. Ozonation Studies 

The HOMF system was evaluated as an ozonation-only setup without the use of membranes. The feed and aqueous O_3_ flux were maintained the same to ensure the same transferred ozone dose (~4 mg L^−1^) as in the filtration experiments. Five different feed solutions were prepared to study how the components of the matrix affected the degradation of the OMPs: (a) OMPs + 1 mM NaHCO_3_, (b) OMPs + 0.05 mM TBA (1:1 molar ratio), (c) OMPs + 0.50 mM TBA (1:10 molar ratio); (d) OMPs + 0.05 mM TBA + 1 mM NaHCO_3_ and (e) OMPs + 0.50 mM TBA + 1 mM NaHCO_3_. Samples for measuring the residual OMPs and ozone concentration were taken over time, as described in Section 2.5. 

### 2.7. Analytical Techniques 

The transferred ozone dose (TOD) was determined by the indigo colorimetric method [43] using a UV-Vis spectrophotometer (Shimadzu UV-1600, Kyoto, Japan) at 600 nm. The OMPs were identified and quantified by high-performance liquid chromatography in reversed-phase mode (HPLC-UV Agilent 1200, Santa Clara, CA, USA). Chromatographic separation was performed using a C18 column (Microsorb, Scituate, MA, USA-MV 100-5 250 × 4.6 mm) at a working temperature of 30 °C and an injection volume of 200 μL. More details on the analytical method are provided in the Supplementary Materials (Text S2). Ion analysis was performed with ion chromatography, total organic carbon (TOC) and total nitrogen (TN) with the TOC analyzer (YSI, Yellow Springs, OH, USA), and metal analysis with ICP-MS Agilent 7500c (Santa Clara, CA, USA). All the samples were filtered via a 0.2 μm nylon filter prior to analysis. 

## 3. Results and Discussion

### 3.1. Ceramic Membrane Modifications and Characterizations

The commercial tubular ceramic membranes purchased by TAMI Industries were modified so they can serve as a substrate for the deposition of metal oxides that will subsequently facilitate the decomposition of aqueous ozone into radicals. The CM with a MWCO of 300 kDa mainly consists of TiO_2_ (support layer) and a thin layer of ZrO_2_ (active layer). The exact phase of the TiO_2_ was unknown; therefore, an XRD analysis was performed. Furthermore, the XRD was applied to the three modified variations of the membranes: (1) one infiltration of cerium oxide (CeO_2_), (2) one infiltration of Ce-doped Ti (CeTiO_x_), and (3) one infiltration of CeO_2_ followed by one infiltration of CeTiO_x_ (CeO_2_ + CeTiO_x_). 

For the diffraction analysis, the tubular membranes were pulverized. Given that the diffraction signal of the specimen is proportional to its weight content, the support layer is the primary contributor to the signal. Consequently, the minor quantities of CeO_2_ or CeTiO_x_ thin films are likely to remain undetected. This is especially true for CeTiO_x_, as its signal would completely overlap with the titania contributions from the substrate when both titania phases share the same polymorph. For this reason, the solid-state tubular membranes were subjected to XRD as a whole body following the Grazing Incidence X-ray Diffraction (GIXRD) configuration with the functional part exposed to radiation. Since the attenuation distance of the X-rays is about a few hundred microns for such materials, it was possible to maximize the collection of the diffraction signal from the upper layers and limit the signal from the bottom material, i.e., the support layer or substrate. A comparison of the XRD and GIXRD results enabled depth profiling.

**Figure 2:**
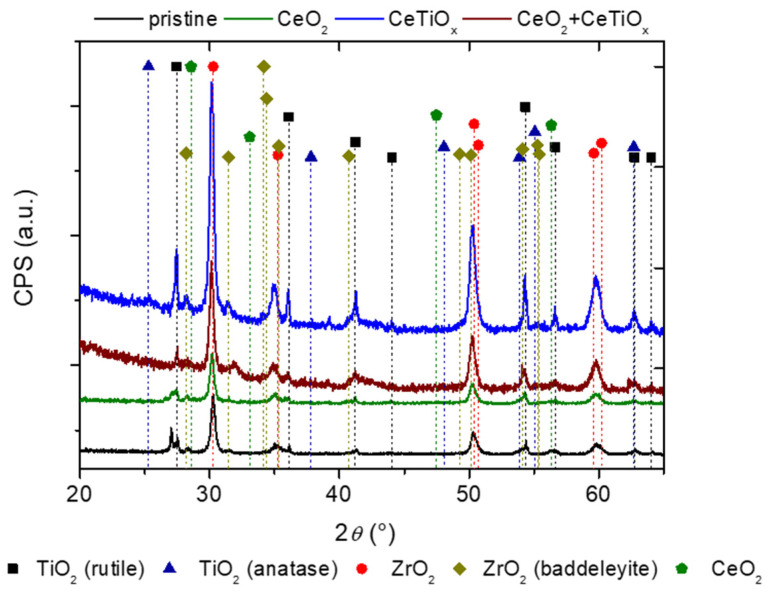
Solid-state X-ray diffraction (SS-XRD) patterns of the modified ceramic membranes pristine (black), CeO_2_ (green) CeTiO_x_ (blue), and CeO_2_ + CeTiO_x_ (red).

XRD (Figure S3) revealed that all the substrates consist of titania rutile (ICDD PDF#21-1276). As alleged, all the other crystalline phases remained undetected. GIXRD analysis (Figure 2) showed that pristine (or unmodified) CM consists of a mixture of titania rutile (ICDD PDF#21-1276) and predominately cubic zirconium oxide (ICDD PDF#49-1642) as the main phase. Due to the splitting of the peaks, the presence of tetragonal zirconium oxide (ICDD PDF#50-1089) is suggested. Also, a baddeleyite zirconia phase (ICDD PDF#37-1484) is present in traces. Furthermore, Figure 2 shows that the main rutile peak splits at about 27°2θ. It is possible to speculate that slightly different rutile phases may be observed from the substrate and from the functional membrane film, confirming that the membrane film can be qualitatively observed.

Regarding the modified membranes, the metal oxides of the nanosized films were identified as titania anatase phase (ICDD PDF#21-1272) and ceria (ICDD PDF#34-0394). Qualitatively, it is quite difficult to differentiate the existence of the titania phases between the substrate and the doped metal oxide as the CeTiO_x_ mainly consists of TiO_2_. Considering the quantitative aspects and previous analysis of the pristine membrane, it is plausible to speculate that the substrate is rutile, as it is unclear what temperatures the commercial substrates and membranes were exposed to. The CeTiO_x_ must then be anatase or brookite, but in borderline detectable quantities. Interestingly, no impurity peaks are evident in the XRD spectra of CeTiO_x_, indicating that the incorporation of Ce ions was successful and did not modify the crystal phase of TiO_2_. This integration likely occurred through either partial substitution of Ti ions or occupation of interstitial sites.

Similarly, from the compositional aspects of the CM, ceria might appear more easily detectable than the titania phases that are present in significant amounts in the substrate. However, this is not the case. Cubic cerium (ICDD PDF#08-0056) and cubic cerium oxide (CeO, ICDD PDF#33-0334) heavily overlap with cubic zirconium oxide (ICDD PDF#49-1642), making them almost undetectable. Despite considerable overlap with baddeleyite zirconia, traces of cerium oxide (CeO_2_, ICDD PDF#34-0394) are observed. 

The catalytic layers of CeO_2_, CeTiO_x_, and CeO_2_ + CeTiO_x_ were deposited onto the surface of the CM (pores and inner surface) by the acid-hydrolyzed sol-gel vacuum infiltration method. Scanning Electron Microscopy (SEM), a powerful imaging technique, was used to visualize the surface morphology and structure of the materials at high resolution. Preliminary trials of multiple deposited layers showed that almost all the triple-layer surfaces have a cracked and uneven appearance, suggesting borderline acceptability for catalysis. For both titania and ceria coatings, it can be roughly generalized that single-layer and sometimes double-layer coatings show acceptable behavior. Based on these results, it was decided to apply up to two layers of catalytic nanomaterials. 

**Figure 3:**
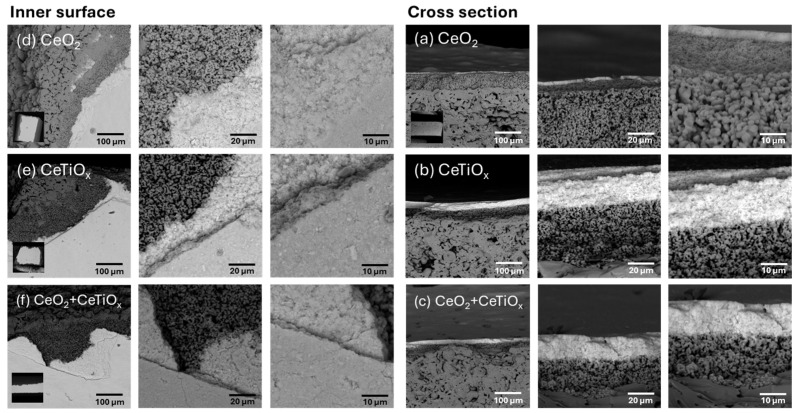
Surface morphologies with Scanning Electron Microscopy (SEM) of the inner surface of the membrane (**left**) and cross-section (**right**) for the three modifications (**a**,**d**) CeO_2_, (**b**,**e**) CeTiO_x_, and (**c**,**f**) CeO_2_ + CeTiO_x_ in various magnifications (50×, 500×, 5000×, and 10,000×).

The surface morphologies of the modified ceramic membranes are displayed in Figure 3. The inner surface of the tubular CMs, where the active layer is located, is shown in Figure 3a–c, while the cross-sectional surface is depicted in Figure 3d–f. The SEM micrographs clearly show three distinct layers: a support layer composed of larger sintered particles, a dense layer composed of medium particles, and an active layer made up of smaller particles. Additionally, all the samples exhibit similar surfaces, characteristic of porous ceramic materials with small pore domains, indicating a well-dispersed distribution of metal oxides on the surface of the CMs. For all the coatings, the majority of the surface appears even, with minimal cracking. This smoothing effect can be attributed to the vacuum infiltration and the drying process prior to calcination. From the SEM pictures, it can be concluded that the active layer of ZrO_2_ is 3–5 μm thick, and the denser layer in between that support and the active layer is around 70–80 μm thick, depending on the area. The thickness of the deposited thin layer of the metal oxides is in the order of nm; however, the SEM resolution could not quantify it.

**Figure 4:**
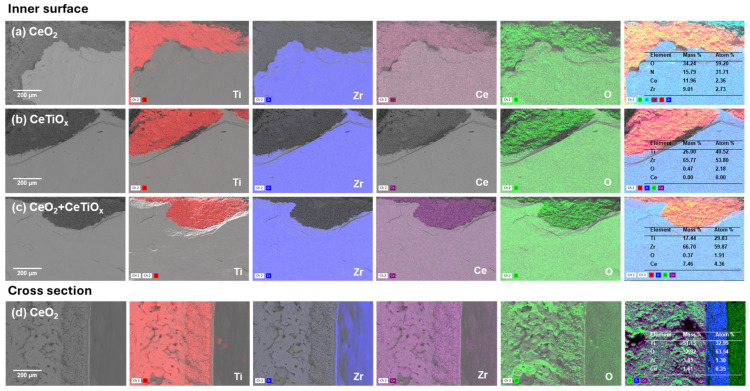
SEM-EDS mapping (250× magnification) of the inner surface of the modified ceramic membranes for (**a**) CeO_2_, (**b**) CeTiO_x_, (**c**) CeO_2_ + CeTiO_x_, and the cross-section for (**d**) CeO_2_.

Additionally, the CMs were characterized using SEM-EDS to determine their elemental composition, with the results shown in Figure 4. The deposited metal oxides on the inner surface of the membrane, as well as the distribution of the elements throughout the support layer, are shown in Figure 4. SEM-EDS analysis revealed that the primary elements are titania (Ti), zirconia (Zr), ceria (Ce), and oxygen (O). The presence of titanium (Ti) is associated with both the support layer and the deposited layer, whereas zirconium (Zr) is linked to the active layer. Oxygen (O) was found in all the samples, as it is a crucial element for forming various oxides. SEM-EDS analysis further confirmed that the layer composed of larger and medium particles is the support layer made of TiO_2_, while the layer consisting of the smallest particles is the active layer made of ZrO_2_. Additionally, the SEM-EDS elemental mappings further confirmed the successful infiltrations of the metal oxides across the CMs’ surface and pores. A higher amount of Ce was found in the CeO_2_ and CeO_2_ + CeTiO_x_ modifications; 11.4 mass % and 7.5 mass % were observed on the surface, respectively. The cross-section surface analysis (Figure 4d) confirmed that Ce atoms are impregnated into the pores of the membrane, confirming the effective use of the vacuum infiltration method. However, after the vacuum infiltration of CMs with CeTiO_x_, Ce was not detected by EDS. In CeTiO_x_, Ti is the dominant element (26% by mass), while Ce is present in a small portion. Therefore, the likely reason Ce was not observed is because it is only present as a doping element in CeTiO_x_ in very low concentrations. The spectra of the EDS analysis are provided in the Supplementary Materials (Figure S4). 

**Figure 5:**
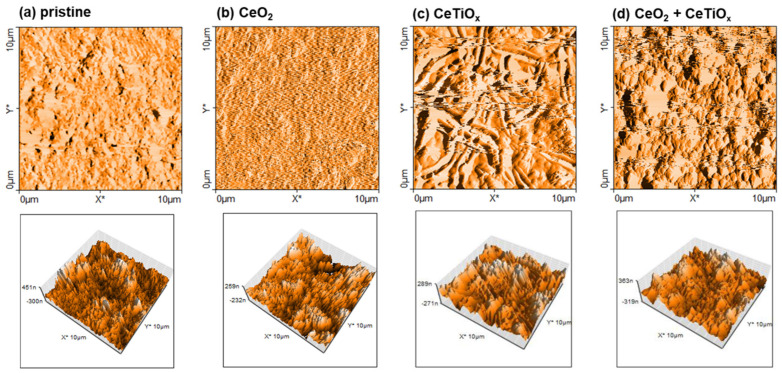
Amplitude (**top**) and three-dimensional (3D) representation (**bottom**) of the surface of the unmodified and modified ceramic membranes: (**a**) pristine, (**b**) CeO_2_, (**c**) CeTiO_x_, and (**d**) CeO_2_ + CeTiO_x_. (X and Y axes represent distance in μm).

Atomic Force Microscopy (AFM) was used to depict the topography and the surface roughness of the unmodified and modified CMs (Figure 5). Unlike optical microscopy, which relies on light waves, AFM operates by physically probing the surface with the tip of the probe, allowing for the visualization of features as small as fractions of a nanometer. AFM analysis showed similar results for all the membranes, and the appearance of the tested samples is attributed to typical porous ceramic materials with small pore domains. The surface roughness (RMS) was 106.4 nm, 110.6 nm, 84.9 nm, and 71.9 nm for pristine, CeO_2_, CeTiO_x_, and CeO_2_ + CeTiO_x_, respectively. The RMS of CeO_2_ modification appeared comparable to that of the pristine CM, indicating that the introduction of CeO_2_ had not significantly affected the membrane surface topography. Conversely, the surface roughness of CeTiO_x_ and CeO_2_ + CeTiO_x_ showed a reduction of 20 and 32%, respectively, compared to the pristine CM, indicating smoother surface topographies, as illustrated in Figure 5c,d. These findings align with the SEM-EDS observations, which confirmed the successful deposition of a smooth CeTiO_x_ catalytic layer on the membrane’s inner surface. We focused purely on the AFM topography, aiming to elucidate the effect of the surface roughness and the homogeneity of the coating on the overall catalytic properties of the membranes.

**Figure 6:**
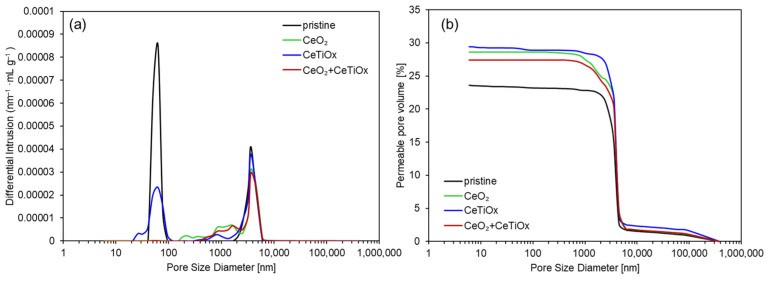
(**a**) Differential intrusion curves and (**b**) permeable pore volume for unmodified and modified ceramic membranes.

Another characterization technique used on the samples was Mercury Intrusion Porosimetry (MIP). MIP is used to determine the pore size distribution and total porosity in solid materials, such as ceramic membranes. Figure 6a demonstrates the pore size distribution (PSD) of each CM specimen, and Figure 6b shows the calculated porosity as a percentage (%). The differential intrusion curves of the pristine and modified CM illustrate that the pores of the membranes were affected by the deposited nanosized material. From Figure 6a, it is apparent that the pristine membrane had more pores below 100 nm, probably ascribed to the active layer of ZrO_2_. Furthermore, the data confirm that the majority of pores in all the CMs were located around 4500 nm, verifying the industrial specifications of CMs, where the support layer (TiO_2_) constitutes a larger proportion. 

The addition of CeO_2_ blocked the smaller pores of the membrane, while the addition of the CeTiO_x_ layer maintained the smaller pores but in a lower quantity. As expected, the double layer of CeO_2_ + CeTiO_x_ showed the lowest number of pores since two layers were deposited on the membrane´s surface, causing further blocking of the small pores. The CeTiO_x_ modification did not obstruct the pore channels because the ionic radii of Ti^3+^/Ti^4+^ (67 and 61 pm) are half the size of Ce^3+^/Ce^4+^ (115 and 101 pm), enabling them to penetrate the pore channels without causing blockages. The fact that the presence of Ce in the CeTiO_x_ did not alter the porosity of the CM suggests that the Ce dopant was seamlessly integrated into the lattice structure, supporting the XRD data.

Figure 6b shows how the permeable pore volume changes with the different dopants. As expected, CeTiO_x_ showed the highest porosity (29.4%), followed by CeO_2_ (28.6%) and CeO_2_ + CeTiO_x_ (27.4%). While it was anticipated that the pristine membrane would exhibit the highest porosity, this was not observed due to the use of a different membrane of the same MWCO for analysis, whereas the other three specimens were derived from the same membrane. However, the important thing is that the pristine membrane had more pores in both ranges, lower than 100 nm (active layer) and 4500 nm (support layer). More detailed data from the MIP analysis are provided in the Supplementary Materials (Table S4). 

### 3.2. Permeability Tests and Properties of the Ceramic Membranes

**Figure 7:**
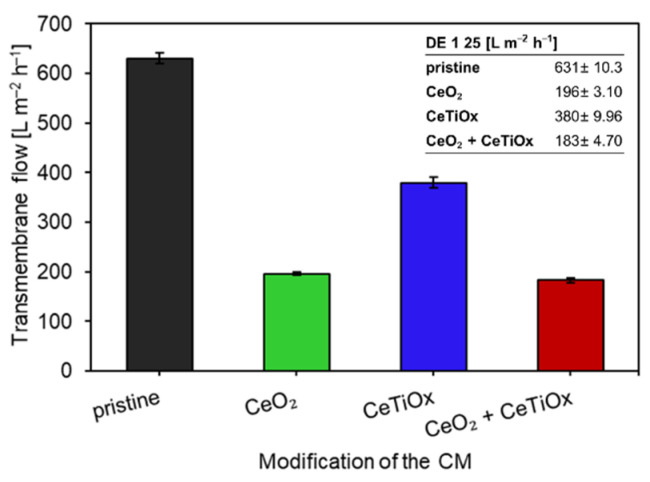
Transmembrane flow of the ceramic membranes at 25 °C and 1 bar (L m^−2^ h^−1^) before and after the deposition of the metal oxides.

Permeability tests were conducted using demineralized water, and the permeability was measured up to six times with good reproducibility (low standard deviation). Figure 7 illustrates the normalized permeability (at 1 bar and 25 °C) of the CMs. The data indicate that the modification of the membranes had a detrimental effect on their permeability, with a 70% reduction observed in the case of the double-layer film comprising CeO_2_ + CeTiO_x_. The notably reduced water permeability of the CeO_2_ + CeTiO_x_ CM is primarily attributed to the additional membrane resistance (*R*_m_) introduced by the CeTiO_x_ catalytic layer. Similarly, the permeability of the CeO_2_-modified CM was reduced by 69%, whereas it dropped by 40% for the CeTiO_x_ modification compared to the pristine membrane. This implies that the permeability of the CeTiO_x_ modification was twice as high as that of the other two modifications. These results are consistent with the MIP analysis, which revealed that the CeTiO_x_-modified CM was the least affected following the deposition of the metal oxide. 

Furthermore, the infiltration of CeO_2_ resulted in pore blockage, as indicated by the MIP analysis, causing a reduction in the size of the support layer pores (Figure 6a; increased pores in the range of 100–1000 nm). Hence, it can be inferred that vacuum infiltration effectively introduced the metal oxides into the pore structure of the CM. However, it did not maintain the permeability unaffected, as reported in the study by Lee et al. (2021) [39]. An intriguing aspect is that the membrane Lee and his collaborators utilized in their experiments operated in a dead-end outside-inside filtration mode, thereby focusing the infiltration on only the exterior surface of the membrane by the dip-coating method. Athanasekou et al. [44] employed a similar method, where they covered both ends of the CM membrane with Teflon tape to prevent the solution from entering the inner part, and the water flux of the modified membrane was even higher than the original. The challenging part in our case was to have the metal oxide deposited on the inner surface of the CMs, and vacuum infiltration was chosen in order to infiltrate the sol-gel into the active and support zone. Consequently, the obstruction of some pores was inevitable. Alternative impregnation methods could be explored to address the loss in water flux. For instance, adopting a layer-by-layer (LbL) assembly method using the dip-coating technique might allow for a more controlled and uniform coating of the active materials. This approach could help optimize the membrane surface properties. However, since the membrane is used in an inside-out filtration mode, the risk of blocking the pores is still high.

Table 1 summarizes the calculated properties of the different CMs. The lowest water permeability and the highest membrane resistance (*R*_m_) were observed in the CeO_2_ + CeTiO_x_ modification, suggesting that the additional layer significantly increased the density and thickness of the membrane (lower porosity than the other two infiltrations). This additional layer likely created a more compact structure, reducing the pore size and increasing the overall water flow resistance, which lowered the permeability. The order for the water resistance was pristine CM > CeTiO_x_ > CeO_2_ > CeO_2_ + CeTiO_x_. 

Furthermore, the hydraulic retention time (HRT) varied among the different CMs. As expected, the pristine CM exhibited the lowest HRT at 8.4 s, indicating a brief contact time between the feed solution and the membrane and, consequently, with ozone. The HRT for the CeTiO_x_ modification was doubled, reaching 18.7 s. Remarkably, the CeO_2_ and the CeO_2_ + CeTiO_x_ modifications demonstrated even higher HRTs, at 34.3 and 35.4 s, respectively, which are four times longer than that of the pristine CM. These elevated HRTs suggest a greater likelihood of the OMPs reacting with ozone, thereby enhancing the catalytic activity occurring within the membrane pores or on the membrane surface. 

### 3.3. Performance Evaluation for the Removal of Pharmaceuticals

The catalytic activity of the pristine and modified CMs was explored in the crossflow hybrid ozonation–membrane filtration (HOMF) process. The feed flow containing the mixture of the OMPs at a concentration of 10 μΜ, as well as the injection flow of aqueous ozone, were kept the same in order to keep the transferred ozone dose (TOD) constant (around 4 mg L^−1^) at the mixing point just before the membrane module. For the evaluation of the process toward the degradation of the pharmaceuticals and to better understand the efficacy of the modified membranes, three series of experiments were performed. The adsorption and ozonation effects were investigated in all of them. In each series, the matrix was different, while the concentration of the OMPs was kept constant. The following matrix was used: (i) demineralized water with bicarbonate, (ii) demineralized water with bicarbonate and scavenger (TBA), and (iii) secondary effluent from Girona WWTP.

The crossflow HOMF system required two minutes to stabilize, so all the degradation data are presented from 2 to 14 min. The operation time was limited to 15 min due to the relatively high feed flow rate of 200 L h^−1^, which was designed to mimic real-world conditions in wastewater treatment plants (WWTPs). Despite the short operational period, the HOMF system maintained stability throughout the experiment.

#### 3.3.1. Effect of Modified Ceramic Membranes on Pharmaceuticals Degradation

**Figure 8:**
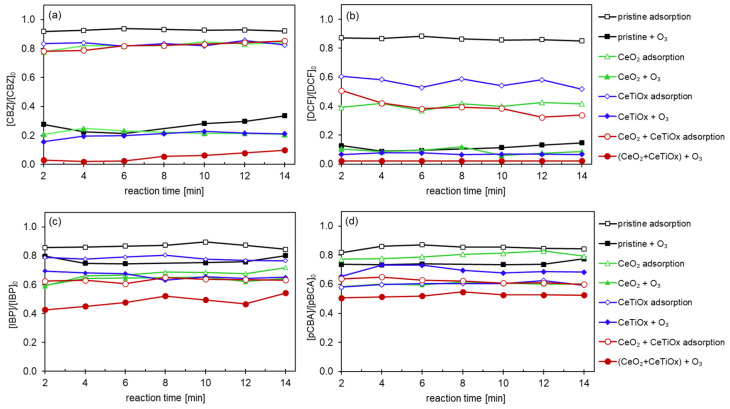
Degradation of the pharmaceuticals (**a**) CBZ, (**b**) DCF, (**c**) IBP, and (**d**) pCBA using the CM 300 kDa MWCO with different surface modifications (Operating conditions: [OMPs]_in_ = 10 μM, [NaHCO_3_] = 1 mM, [TOD] = 4 mg L^−1^).

Figure 8 displays the catalytic degradation of CBZ, DCF, IBP, and pCBA by different CMs. CBZ was barely adsorbed on the surface of the pristine CM. In contrast, the three modified membranes demonstrated similar adsorption capacities, reducing the concentration of CBZ by 20%. Interestingly, DCF was adsorbed by the modified membranes, reaching a 60% reduction when CeO_2_ and CeO_2_ + CeTiO_x_-modified CM were used and a 40% reduction when CeTiO_x_ CM was utilized. Similarly, IBP and pCBA showed the lowest degradation by adsorption when the pristine CM was used and up to 40% by the modified CMs. For IBP (Figure 8c), the highest degradation by adsorption was observed in CeO_2_ + CeTiO_x_, followed by CeO_2_, CeTiO_x_, and finally, by the pristine CM. For pCBA (Figure 8d), the order of the adsorption efficiency was CeO_2_ + CeTiO_x_ = CeTiO_x_ > CeO_2_ > pristine CM. 

The enhanced ability of the modified membranes to adsorb DCF, IBP, and pCBA can be attributed to the deprotonated state of these compounds in the treated solution, as their *pK*a values are lower than the pH of the solution (*pK*a < pH; Table S1). This deprotonation increases the negative charge on the molecules, which likely enhances their interaction with the positively charged or adsorptive sites on the modified membrane surfaces. Consequently, this electrostatic attraction facilitates more efficient adsorption of these compounds compared to their protonated forms. Furthermore, this explains the low adsorption capacity of CBZ, which remains in its protonated form and, thus, does not interact effectively with the deposited metal oxides on the modified membranes.

When an aqueous ozone (O_3_(aq)) was introduced into the system, the degradation efficiency of the organic micropollutants (OMPs) improved across all the treatments. Specifically, CBZ showed up to 80% degradation with the pristine, CeO_2_-, and CeTiO_x_-modified ceramic membranes (CMs). Complete mineralization of CBZ was achieved with the double-layer modification. This trend was similarly observed for DCF, with the pristine and monolayer modifications achieving 90% degradation.

In comparison, IBP and pCBA, known for their resistance to ozone (*k*_O3/IBP_ = 9.6 M^−1^ s^−1^ and *k*_O3/pCBA_ < 0.15 M^−1^ s^−1^), exhibited the highest degradation rates with the CeO_2_ + CeTiO_x_ modification. This result suggests the catalytic decomposition of ozone into hydroxyl radicals (^•^OH), leading to nearly 50% degradation of these recalcitrant compounds. The presence of ^•^OH significantly enhanced the degradation of both CBZ and DCF. The additional CeTiO_x_ layer proved to be an effective filtration and catalytic layer, further improving the performance.

CBZ and DCF are more readily degraded compared to IBP and pCBA due to their higher reactivity with ozone (*k*_O3/CBZ_ = 3.00 × 10^5^ M^−1^ s^−1^, *k*_O3/DCF_ = 6.85 × 10^5^ M^−1^ s^−1^). The higher rate constants for ozone reactions with CBZ and DCF explain their more efficient degradation under ozonation treatments. This observation aligns with findings in the literature, where compounds with electron-rich functional groups react more readily with ozone, whereas those with electron-withdrawing groups, such as IBP and pCBA, show reduced reactivity and require additional catalytic mechanisms to enhance the degradation [45].

#### 3.3.2. Effect of Scavenger on Pharmaceuticals Degradation 

**Figure 9:**
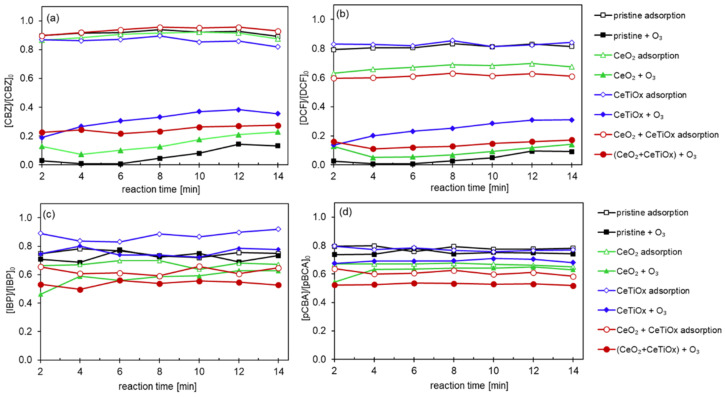
Degradation of the pharmaceuticals (**a**) CBZ, (**b**) DCF, (**c**) IBP, and (**d**) pCBA using the CM 300 kDa MWCO with different surface modifications in the presence of TBA, a hydroxyl radical scavenger at 1:1 molar ratio relative to the total OMPs (Operating conditions: [OMPs]_in_ = 10 μM, [TBA] = 0.05 mM, [NaHCO_3_] = 1 mM, [TOD] = 4 mg L^−1^).

The following series of experiments incorporated tertiary butanol (TBA) as a hydroxyl radical scavenger at a molar ratio of 1:1 (0.05 mM TBA) relative to the total concentration of OMPs. This approach allowed us to isolate the effect of the surface modifications on the degradation process. Particularly, the removal efficiencies of CBZ and DCF (Figure 9a,b) in the presence of the TBA scavenger followed the order of pristine CM > CeO_2_ > CeO_2_ + CeTiO_x_ > CeTiO_x_, highlighting the influence of the membrane surface modifications on the degradation performance under these specific conditions. 

The fact that the pristine membrane revealed the best degradation for the compounds easily degraded by ozone suggests that the short HRT in combination with TBA enhanced the direct oxidation with ozone as the produced ^•^OH reacted with the TBA. More specifically, the short contact time limited the catalytic activity at the membrane’s inner surface, only favoring the compounds that can be easily degraded by ozone. Again, the CBZ was barely adsorbed onto the CM’s surface, while DCF exhibited higher degradation (up to 40% with CeO_2_ + CeTiO_x_) due to the electrostatic interactions with the deposited metal oxides. 

The CeO_2_ and CeO_2_ + CeTiO_x_-modified CMs achieved high degradation rates for IBP and pCBA, up to 40–50% (Figure 9c,d). These findings emphasize the importance of CeO_2_ infiltration throughout the microporous structure of the CMs for effective catalytic degradation and mineralization of IBP and pCBA, significantly improving access to catalytic sites. Additionally, the CM pores can limit reactants within the microreactor environment, enhancing the interphase mass transfer and catalytic performance [46]. Among the different modified CMs studied, CeO_2_ + CeTiO_x_ stands out as ideal for hybrid process applications, with the CeTiO_x_ catalytic layer coating the membrane’s inner surface and CeO_2_ infiltration throughout the membrane’s substrate. This functionalization enables catalytic effects on both the membrane surface and internal pore surfaces. Consequently, using CeO_2_ + CeTiO_x_ in this hybrid system can create a synergistic effect between membrane filtration and catalytic ozonation, leading to the effective degradation and mineralization of OMPs.

The ease or difficulty of degrading an OMP with ozone depends on its chemical structure and functional groups. Due to its electronic configuration, ozone can engage in various reactions in water, including oxidation, cycloadditions, and electrophilic substitutions [47]. Compounds that are easily degradable typically possess electron-rich functional groups, which readily undergo electrophilic substitution with ozone. Such functional groups include double bonds (C=C; found in CBZ), tertiary amines (^3^C-NH_2_), aniline (−C−NH_2_; found in DCF), phenol (C_6_H_5_OH), and methoxy groups (−O−CH_3_) [45,48,49,50,51].

On the other hand, OMPs that are more challenging to remove often contain electron-withdrawing groups, such as fluoro (–C–F), nitro (–O–N=O), chloro (–C–F; found in pCBA), amide (−C(=O)−NH_2_), and carboxyl (–C(=O)OH; found in IBP, and pCBA) [45,50]. These groups decrease the electron density in the compound, inhibiting the electrophilic substitution reactions. Additionally, the electronegative nature of these groups makes them less reactive to ozone, creating a shielding effect.

Remarkably, some OMPs, like carbamazepine and diclofenac, remain reactive with ozone despite containing electron-withdrawing groups (amide in CBZ; chloro, and carboxylic acid in DCF). This suggests that the presence and position of high-electron-density functional groups, such as the aromatic amine in DCF and the C=C double bond in CBZ [45], can counteract the inhibitory effects of the electron-withdrawing groups. Ibuprofen, which lacks electron-rich functional groups, is resistant to ozone treatment. However, it can be effectively removed through intensive biological treatment [52,53]. Additionally, ozone-recalcitrant micropollutants (e.g., atrazine or pCBA) can potentially be removed through oxidation involving hydroxyl radicals [54].

#### 3.3.3. Effect of Matrix on Pharmaceuticals Degradation Only with Ozonation 

**Figure 10:**
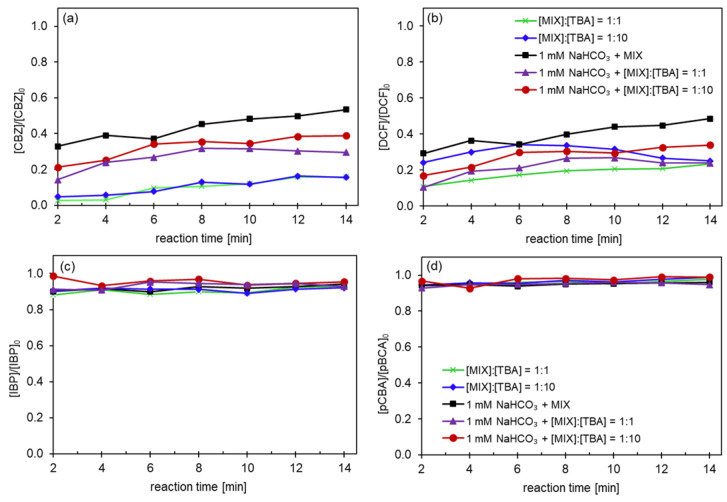
Degradation of the pharmaceuticals (**a**) CBZ, (**b**) DCF, (**c**) IBP, and (**d**) pCBA with ozonation alone in the presence of bicarbonate and/or scavenger (Operating conditions: [OMPs]_in_ = 10 μM, [TBA] = 0.05 mM (1:1) or 0.5 mM (1:10), [NaHCO_3_] = 1 mM, [TOD] = 4 mg L^−1^).

Experiments using the HOMF system without any ceramic membranes were conducted to investigate the effects of ozonation alone. Five different matrices were tested to determine, first, whether the presence of bicarbonate (1 mM NaHCO_3_) influences the decomposition of O_3(aq)_ into ^•^OH, and second, how the concentration of a scavenger impacts the degradation of organic micropollutants (OMPs) by ozone. Figure 10 shows the degradation of the four model compounds when only ozonation is applied under consistent operational conditions. As expected, the hydraulic retention time (HRT) was identical for all the runs, limited to 0.56 s. This short HRT indicates a challenge in achieving the decomposition of O_3_ into ^•^OH, resulting in inadequate degradation of IBP and pCBA. 

Additionally, the CBZ and DCF degradation is primarily determined by the integral of the ozone exposure; the higher the exposure, the higher the degradation. From Figure 10a,b, it can be seen that the presence of TBA increased the degradation of the CBZ up to 90%, irrespective of its concentration, as it reacts with the potential radicals, thereby increasing the exposure of the OMPs to ozone. Similarly, DCF degraded more in the presence of TBA, although when bicarbonate was also added to the matrix, the degradation slightly decreased. The lower degradation for both compounds CBZ and DCF (~65%) was recorded when the mixture (MIX) of the OMPs was exposed to ozone in the presence of bicarbonate. This is because bicarbonate acts as a radical scavenger, reducing the availability of ^•^OH radicals for further degradation of the OMPs. The experiments underscore the significance of the retention time and matrix composition in the effectiveness of ozonation for OMP degradation. 

#### 3.3.4. Application of Real WWTP Secondary Effluent on HOMF System

The crossflow HOMF system was further tested with secondary effluent (SE) obtained from the WWTP of Girona (Catalonia, Spain) to evaluate the effect of a complex water matrix on the hybrid process. The CeO_2_ + CeTiO_x_-modified membrane, which demonstrated the best performance in removing the four OMPs, was selected for these experiments, and its performance was compared to the pristine CM. Both membranes underwent a four-cycle filtration operation with cleanings between cycles using 0.5 M NaOH and demineralized water. During the first two cycles, the adsorption effect was assessed, while aqueous O_3_ was introduced in the latter two cycles. The permeability of the membranes was measured after each cleaning step, and subsequent experiments were not conducted until the permeability was restored (up to four times cleaning). The use of a complex matrix such as SE was expected to result in membrane fouling, i.e., lower permeability after each run. 

**Figure 11:**
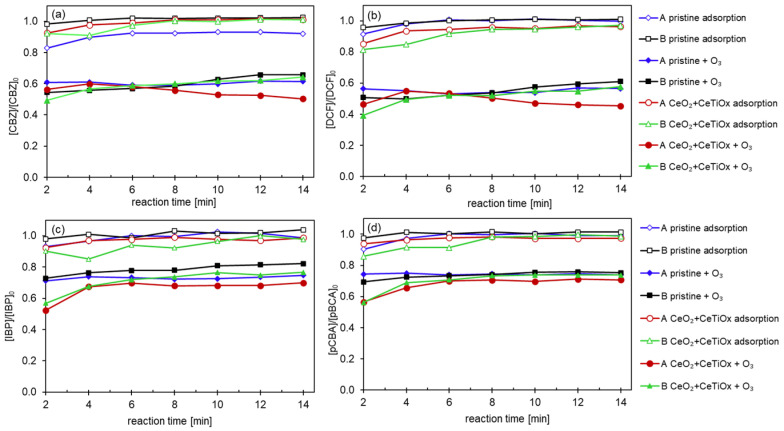
Degradation of the pharmaceuticals (**a**) CBZ, (**b**) DCF, (**c**) IBP, and (**d**) pCBA using the pristine and CeO_2_ + CeTiO_x_ CM 300 kDa MWCO in secondary effluent (Operating conditions: [OMPs]_in_ = 10 μM, [TOD] = 4 mg L^−1^).

Figure 11 illustrates the degradation of the four OMPs after either adsorption or ozonation using the pristine and CeO_2_ + CeTiO_x_-modified CMs. The results for the four cycles are presented separately to clearly demonstrate the performance of each experimental run. Notably, only CBZ exhibited a significant change in degradation during adsorption using the pristine CM; the degradation was negligible in the first cycle but increased to 15% in the second cycle. For DCF, IBP, and pCBA, the adsorption levels remained consistent across treatments, following similar trends. While the adsorption effect was more pronounced in the experiments with clean water, it is believed that the numerous compounds and ions found in SE competed for electrostatic interactions with the tested OMPs, thereby diminishing the adsorption efficiency.

Regarding ozonation, CBZ and DCF demonstrated consistent degradation rates of approximately 50% across all the runs, with no significant variations between cycles. In contrast, IBP and pCBA exhibited lower degradation rates of around 30%. However, the modified CeO_2_ + CeTiO_x_ membrane showed slightly improved performance compared to the pristine membrane. These results confirm that the impregnated CM can maintain its catalytic activity even in complex matrices like secondary effluent, enhancing the degradation of OMPs despite the presence of competing compounds and ions. Undeniably, the removal efficiency for all the OMPs was significantly lower than that of the DI water (CBZ and DCF were almost mineralized) as the molecules would not compete for ozone or radicals. These findings are in line with the results of Lee et al. (2021) [39], where DEET was degraded by ~25% in RO-rejected water when the modified CM was utilized. This further highlights the potential of CeO_2_ + CeTiO_x_-modified membranes in advanced wastewater treatment processes where diverse and challenging water matrices are encountered. 

Table 2 summarizes the chemical characteristics of the SE and the permeate collected from the adsorption and ozonation experiments. Notably, the total organic carbon (TOC) of the SE doubled after the addition of the OMPs. Adsorption experiments with the pristine CM did not reduce the organic load; however, a decrease from 29.5 to 23 mg L^−1^ was observed when O_3(aq)_ was applied. The CeO_2_ + CeTiO_x_-modified CM showed a lower TOC during adsorption compared to the pristine CM, and an even lower TOC of 20.2 mg L^−1^ during ozonation.

These results indicate that the degradation of OMPs can lead to an increase in the TOC, as noted in previous studies [39], and that ozonation in complex matrices can slightly decrease the organic load. Although a higher ozone dose would likely be more effective, it would also increase the process cost. Additionally, Table 2 shows that the concentrations of the measured anions and cations were lower with the CeO_2_ + CeTiO_x_ treatment, suggesting ion rejection phenomena. This is further supported by a ~30% reduction in conductivity. Thus, it can be confirmed that the two-layer modification not only promotes the decomposition of the O3 but can also increase the size exclusion of the CM by adsorbing even small inorganic contaminants (bromate was not detected in the sample analysis). 

**Figure 12:**
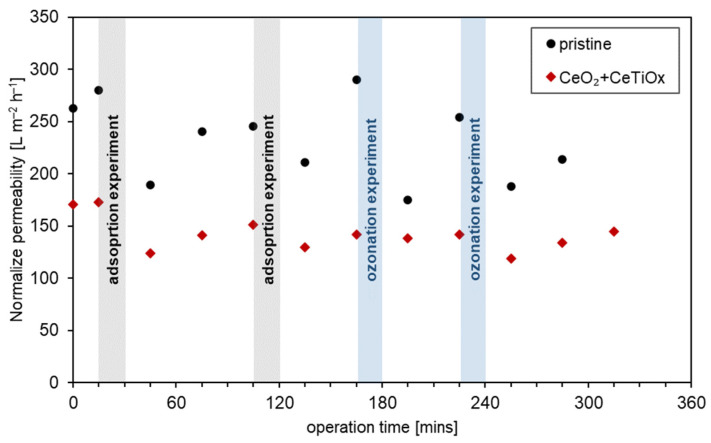
Permeability recovery after adsorption and ozonation experiments with secondary effluent for pristine (black dots) and CeO_2_ + CeTiO_x_-modified (red diamonds) ceramic membrane.

During the four-cycle experiments, the permeability or transmembrane flow (L m^−2^ h^−1^) was recorded (Figure 12). The initial permeability of the CMs was measured before starting the experiments using DI water, and it was found to be 280 and 173 L m^−2^ h^−1^ for the pristine and CeO_2_ + CeTiO_x_ CM, respectively. After the first adsorption experiment, the permeability dropped by 33% for the pristine, while a 29% reduction was recorded for the CeO_2_ + CeTiO_x_ modification. The CMs were washed with 0.5 M NaOH, followed by three cleanings using DI water. The permeability was partially recovered, and the second adsorption experiment was performed. Again, the permeability was reduced at the end of the experiment by 15% for both membranes. The same cleaning procedure was repeated, and the ozonation experiment was performed. Notably, the permeability of the pristine membrane was fully recovered, reaching 290 L m^−2^ h^−1^. Regarding the modified CM, the permeability was recovered when compared to the beginning of the second cycle, but it failed to reach the initial permeability value.

Interestingly, the permeability of the pristine membrane dropped by 33% after the first ozonation experiment, suggesting fouling, likely due to pore blockage. However, after the second cleaning, the membrane fully recovered its permeability. In contrast, the double-layer modified CM did not exhibit a significant decrease in permeability after the ozonation experiment, indicating that the catalytic layer provided antifouling properties in the presence of O_3_. In the fourth and final cycle, the permeability decreased by 26% for the pristine CM and 17% for the modified CM, reaffirming the effectiveness of the deposited metal oxides in degrading OMP molecules into smaller compounds. After repeating the cleaning procedure, the permeability was restored to its level before the second cycle. Nonetheless, some pores were inevitably permanently blocked, and a thermal treatment would likely be necessary to return the CM to its initial state.

During the experiments, samples from the permeate were collected to test whether there was any leaching of the metals (Ce or Ti) occurring after their deposition. Metal analysis revealed that no leaching occurred as the Ce^3+^/Ce^4+^ and Ti^3+^/Ti^4+^ concentrations were under the detection limit. 

**Figure 13:**
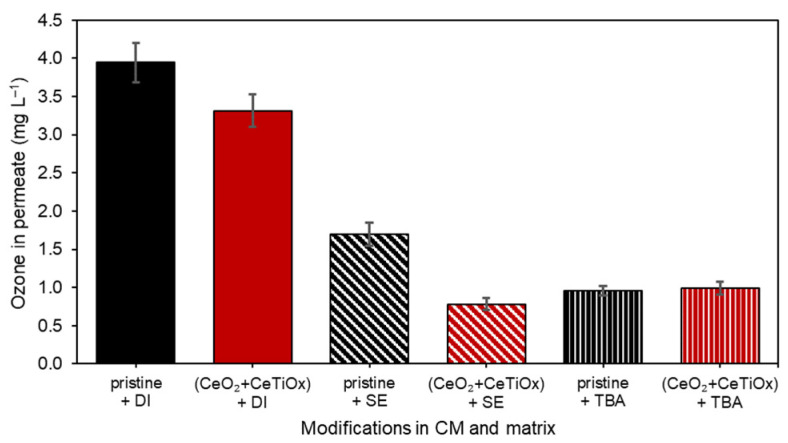
Residual ozone concentration [mg L^−1^] in permeate flow for pristine and CeO_2_ + CeTiO_x_-modified membrane with demineralized water, with TBA at 1:1 molar ratio and Girona WWTP secondary effluent (ozone residual concentration was determined with the indigo method at 600 nm).

Figure 13 illustrates the ozone concentration in the permeate during the HOMF process using a pristine and a modified CM with CeO_2_ + CeTiO_x_. Three different matrices were tested: (a) demineralized water (DI) with bicarbonate, (b) DI with the addition of TBA as a hydroxyl radical scavenger, and (c) secondary effluent (SE). Figure 13 shows that the pristine CM with DI water exhibited the highest ozone concentration in the permeate, approximately 4 mg L^−1^. This high concentration suggests minimal ozone decomposition or reaction with the membrane and matrix, probably due to the low HRT. The modified CM also showed a high ozone concentration in the permeate, slightly lower than the pristine CM but still around 4 mg L^−1^. The slight reduction indicates some catalytic activity of the CeO_2_ + CeTiO_x_ layer, contributing to ozone decomposition.

When TBA, a hydroxyl radical scavenger, was added, the ozone concentration in the permeate dropped significantly to around 1 mg L^−1^. This decrease suggests that TBA effectively scavenged ^•^OH, reducing the ozone decomposition and thus lowering the permeate concentration. Similar to the pristine CM, the ozone concentration with the modified CM and TBA also dropped to around 1 mg L^−1^. This indicates that the presence of the CeO_2_ + CeTiO_x_ layer did not significantly change the scavenging effect of TBA on ^•^OH. In the SE matrix, the ozone concentration in the permeate was lower than in the DI water matrix, around 2 mg L^−1^. This suggests that the more complex composition of SE, including various organic and inorganic compounds, contributed to the higher ozone consumption. The modified CM in SE showed an ozone concentration of approximately 1.5 mg L^−1^, which is lower than the pristine CM in SE. This indicates the enhanced catalytic activity of the CeO_2_ + CeTiO_x_ layer in the more complex SE matrix, leading to greater ozone decomposition and reduced permeate concentration. Moreover, this can be attributed to the higher HRT since the ozone has more time to enter the pores and decompose. 

The results demonstrate that both the pristine and modified CMs show high ozone concentrations in simpler matrices (DI water), with slightly better performance by the pristine CM. The addition of TBA significantly reduces the ozone concentration, highlighting its effectiveness in scavenging hydroxyl radicals. In the complex SE matrix, the ozone concentrations are generally lower, with the modified CM exhibiting better catalytic performance in decomposing ozone compared to the pristine CM. This suggests that the CeO_2_ + CeTiO_x_ layer enhances the catalytic ozonation process, particularly in complex matrices, making it more effective for wastewater treatment applications where ozone decomposition and OMP degradation are crucial.

Utilizing the HOMF process with an inside-out configuration and tubular ceramic membranes at a 200 L h^−1^ flow rate demonstrates a high degree of universality and applicability for real-world WWTPs. This approach combines the oxidative strength of ozonation, which breaks down complex contaminants, with the physical separation capabilities of membrane filtration, resulting in high-quality effluent. In addition, tubular ceramic membranes are known for their robustness and long operational life, making them particularly suitable for sustainable WWTP operations. Additionally, their catalytic modification can enhance the contaminant retention within the membrane, reducing fouling and increasing the overall efficiency. 

Studies such as those by Chen et al. [55] and Psaltou et al. [13] have demonstrated significant improvements in contaminant removal when combining multiple treatment technologies, similar to the hybrid approach used in this study. Furthermore, the research by Kisielius et al. [56] on ozonation followed by granular activated carbon (GAC) filtration and the recent review by Xie et al. [57] highlighted the advantages of integrating ozonation with filtration. In this study, ceramic membranes offer a more durable and fouling-resistant alternative, enhancing both the efficiency and sustainability of the treatment process. These findings confirm that the HOMF methodology with tubular catalytic ceramic membranes is a robust and effective solution for advanced wastewater treatment, capable of addressing a wide range of contaminants while maintaining operational stability.

Interestingly, Eniola et al. [7], in their review of conventional and advanced hybrid technologies, concluded that hybrid technologies have demonstrated greater effectiveness than individual methods. However, one of the drawbacks of such technologies is the required pretreatment when the matrices are complex and the need for additional treatment units, thus increasing the operational cost. The main operational cost of the HOMF system was calculated to be EUR 1.135 m^−3^ of treated water, EUR 0.755 m^−3^ for the centrifugal pump, and EUR 0.38 m^−3^ for the ozone generator. Yet, the cost is lower than those of the advanced oxidation processes using UV reactors, which can reach more than EUR 10 m^−3^ [58].

By mimicking actual WWTP conditions, this study ensured that its findings are relevant and applicable, supporting the scalability of this hybrid system for both small-scale pilot studies and large-scale implementations.

## 4. Conclusions

The vacuum infiltration method for the deposition of metal oxides using the sol-gel technique proved to be highly effective, especially when there is an inside-out filtration mode. Both CeO_2_ and CeTiO_x_ were successfully infiltrated through the membrane pores, resulting in a well-distributed and stable modification layer. SEM-EDS analysis showed a good distribution on the surface and inside the pores, with 11.96% and 1.61% mass of CeO_2_, respectively. This method ensured that the metal oxides were thoroughly embedded within the membrane structure, enhancing the catalytic properties of the membranes and contributing to their overall performance.

Besides oxidation by ozone, pharmaceuticals were removed through rejection and adsorption, which was significant with the double surface modification of the membrane. DCF, IBP, and pCBA were rejected by 60%, 40%, and 40%, respectively. This can be attributed to the interaction between the membrane surface and these compounds. In contrast, the adsorption removal of CBZ (20%) was less effective, likely due to its hydrophilic properties, which reduce its interaction with the hydrophobic membrane surface, leading to lower adsorption efficiency.

Introducing aqueous ozone greatly enhanced the degradation of organic micropollutants (OMPs). The modified membranes exhibited varying levels of efficiency in degrading these compounds. CBZ and DCF were more readily degraded compared to IBP and pCBA (>90% and <50%, respectively) due to their higher affinity for ozone, as evidenced by their higher reaction rate constants. This indicates that the chemical properties of these compounds play a crucial role in their degradation efficiency when exposed to ozone. Among the modifications, the CeO_2_ + CeTiO_x_ coating was particularly effective. This modification facilitated the generation of hydroxyl radicals (^•^OH), which are highly reactive and capable of degrading ozone-sensitive and ozone-resistant compounds. Consequently, this led to improved degradation performance across a range of OMPs.

Finally, the double-layer modification with CeO_2_ + CeTiO_x_ demonstrated the highest degradation efficiency of all the experimental conditions. Notably, this modification achieved nearly complete mineralization of CBZ and DCF, showcasing its superior catalytic performance. However, when used in complex matrices (WWTP secondary effluent), the degradation decreased from 95–98% to 50% due to the competition with other compounds. 

## Figures and Tables

**Table 1:** Properties of the unmodified and modified CMs.

CM	Water Permeability, L m^−2^ h^−1^	Porosity (*ε*)	HRT, s	*R*_m_, ×10^−10^ m^−1^	Surface Roughness, nm
Pristine	631	0.22	8.4	0.39	106.4
CeO_2_	196	0.29	34.3	1.23	110.6
CeTiO_x_	360	0.29	18.7	0.65	84.9
CeO_2_ + CeTiO_x_	183	0.27	35.4	1.31	71.9

**Table 2:** Chemical characteristics of the secondary effluent and the permeate from the adsorption and ozonation experiments using the pristine and the CeO_2_ + CeTiO_x_-modified ceramic membrane.

Parameter	Units	Secondary Effluent	Pristine		CeO_2_ + CeTiO_x_	
			Adsorption	Ozonation	Adsorption	Ozonation
TOC	mg L^−1^	13.89 ± 1.26	29.47 ± 0.46	22.99 ± 1.08	24.12 ± 0.88	20.26 ± 0.90
TN	mg L^−1^	3.14 ± 0.13	3.02 ± 0.01	2.52 ± 0.14	2.65 ± 0.06	2.22 ± 0.08
N-NO_3_	mg L^−1^	1.36 ± 0.33	1.83 ± 0.03	1.31 ± 0.10	1.55 ± 0.02	1.25 ± 0.01
N-NO_2_	mg L^−1^	0.07 ± 0.03	0.03 ± 0.00	0.06 ± 0.00	0.04 ± 0.00	0.04 ± 0.01
N-NH_4_	mg L^−1^	0.01 ± 0.00	0.02 ± 0.01	0.01 ± 0.00	0.03 ± 0.02	0.01 ± 0.00
S-SO_4_	mg L^−1^	21.63 ± 0.05	18.70 ± 0.37	19.21 ± 0.32	18.97 ± 0.18	18.13 ± 1.10
Na	mg L^−1^	111.2 ± 0.60	99.2 ± 0.05	104 ± 0.60	101.2 ± 1.54	100.2 ± 6.20
Ca	mg L^−1^	73.64 ± 0.23	64.21 ± 1.36	66.97 ± 1.25	60.35 ± 2.25	54.69 ± 6.26
K	mg L^−1^	22.10 ± 0.16	18.98 ± 0.29	19.37 ± 0.37	18.91 ± 0.17	18.23 ± 1.07
Mg	mg L^−1^	12.43 ± 0.06	11.04 ± 0.17	11.62 ± 0.22	11.15 ± 0.12	10.66 ± 0.70
F	mg L^−1^	0.12 ± 0.01	0.10 ± 0.00	0.11 ± 0.01	0.10 ± 0.05	0.06 ± 0.01
Cl	mg L^−1^	153.9 ± 0.09	135.9 ± 2.69	143.5 ± 2.23	139.2 ± 1.30	133.5 ± 8.83
Conductivity	μS cm^−1^	1016 ± 55.7	908.5 ± 6.5	865.5 ± 48.5	790 ± 102	749 ± 33
pH		7.68 ± 0.07	7.86 ± 0.15	8.22 ± 0.12	7.81 ± 0.06	7.87 ± 0.08

Values are presented as the average ± standard deviation (SD), n = 4 for SE and n = 2 for permeate. Values for the treatments correspond to the concentration of the measured parameter in permeate.

## Data Availability

All data can be accessed upon request to the corresponding author.

## Supplementary Materials

The following supporting information can be downloaded at https://www.mdpi.com/article/10.3390/nano14131163/s1, Table S1: Physicochemical characteristics of the model organic micropollutants; Table S2: Characteristics of the tubular ceramic membrane (TAMI Industries, France); Table S3: HPLC-UV mobile phase; Table S4: Parameters provided by the Mercury Intrusion Porosimeter for the tested ceramic membranes; Text S1: Details on the preparation of the modified ceramic membranes; Text S2: HPLC method for the qualification and quantification of the model compounds; Figure S1: Scheme for the impregnation of the ceramic membranes with the vacuum impregnation technique; Figure S2: Custom-built crossflow filtration unit with an inside-out configuration (SIVA, France). Figure S3: XRD analysis of the pulverized ceramic membranes before and after modifications (left) and XRD analysis of the deposited nanoparticles on a conductive glass to remove the noise of the background (i.e., ceramic membrane); Figure S4: EDS spectra of the inner surface of the modified ceramic membranes for (a) CeO_2_, (b) CeTiO_x_, (c) CeO_2_ + CeTiO_x_, and the cross-section for (d) CeO_2_.
