# Supplementary material for: Catalytic Ozonation of Pharmaceuticals Using CeO2-CeTiOx-Doped Crossflow Ultrafiltration Ceramic Membranes
Source: Nanomaterials (Basel). 2024 Jul 7;14(13):1163. doi: 10.3390/nano14131163 (PMC11243686; doi:10.3390/nano14131163)
Supplement: Supplementary file 1 [file nanomaterials-14-01163-s001.zip › nanomaterials-3050450-supplementary.pdf]

Supplementary material

Catalytic Ozonation of Pharmaceuticals using CeO_2-$CeTiO_x$ doped Crossflow Ultrafiltration Ceramic Membranes

Nikoletta Tsiarta[1,2,3], Silvia Morović[4], Vilko Mandić[4], Ivana Panžić[4], Roko Blažic[4], Lidija Ćurković[3], and Wolfgang Gernjak[1,5]*

[1] Catalan Institute of Water Research, Carrer Emili Grahit 101, 17003 Girona, Spain; ntsiarta@icra.cat
[2] University of Girona, Campus de Montilivi, 17003 Girona, Spain
[3] Faculty of Mechanical Engineering and Naval Architecture, University of Zagreb, Ivana Lučića 5, 10002 Zagreb, Croatia; lidija.curkovic@fsb.unizg.hr (L.Ć.)
[4] Faculty of Chemical Engineering and Technology, University of Zagreb, 10000 Zagreb, Croatia; smorovic@fkit.unizg.hr (S.M.), vmandic@fkit.unizg.hr (V.M.), ipanzic@fkit.unizg.hr (I.P.), rblazic@fkit.unizg.hr (R.B.)
[5] Catalan Institution for Research and Advanced Studies (ICREA), 08010, Barcelona, Spain; wgernjak@icra.cat
* Correspondence: wgernjak@icra.cat (W.G)

The following information supports the text of the manuscript.

Table S1. Physicochemical characteristics of the model organic micropollutants

**Organic Micropollutant**	**Molecular structure**	**Molar mass, g mol⁻¹**	**Log *Kow***	***pKa***	***k*$_{O3}$ M^{-1} s^{-1}**	***k*$_{\bullet OH}$ M^{-1} s^{-1}**
Carbamazepine (CBZ)	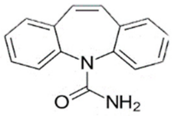	236.27	2.45	13.9	3.00×10^5	8.80×10^9
Diclofenac (DCF)	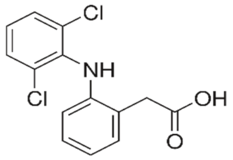	296.15	4.51	4.15	6.85×10^5	7.50×10^9
Ibuprofen (IBP)	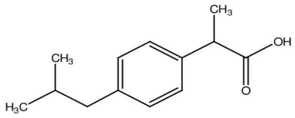	206.29	3.97	4.4	9.6	7.40×10^9
para-chloro benzoic acid (pCBA)	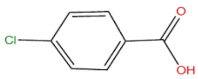	156.57	2.65	3.98	< 0.15	5.00×10^9

Table S2. Characteristics of the tubular ceramic membrane (TAMI Industries, France)

**Parameter**	**Description**
Molecular Weight Cut-Off (MWCO)	300 kDa
Filtration range	Ultrafiltration
Support material	Titania Oxide (TiO_2)
Average pore diameter	4500 nm
Active material	Zirconia Oxide (ZrO_2)
Pore size of active layer	70 – 80 nm
Length	250 mm
External diameter	10 mm
Internal diameter	6 mm
Operation	Inside-out

Text S1. Details on the Preparation of modified ceramic membranes

Before infiltration, the ceramic membranes were thoroughly cleaned with 0.5 M NaOH and deionized (DI) water using an ultrasonic bath, and then allowed to dry at room temperature. The cleaned membranes were placed into a glass tube within a sealed filtration flask connected to a vacuum pump. The vacuum pump was activated to evacuate all air from the flask, enabling the membrane pores to be infiltrated by the sol (colloidal solution). The membranes were subjected to a vacuum of 0.2 bar for 15 minutes. Sol (colloidal solution) was then gradually introduced from the top of the filtration flask, allowing it to completely cover the membranes. The setup is illustrated in Figure S1.

The membranes remained submerged in the sol-gel under vacuum conditions for 20 minutes to ensure thorough infiltration (Figure S1). Following this, the membranes were removed from the sol and positioned vertically on a stand to dry. To further enhance oxide dispersion and drying, the membranes were returned to the vacuum environment at 0.2 bar for an additional 30 minutes. Post-vacuum treatment, the membranes were placed in an oven at 90 °C for 15 hours to age, followed by drying at 120 °C for 3 hours. Finally, the membranes underwent calcination, with CeO_2 treated at 400 °C for 5 hours and $CeTiO_x$ at 450 °C for 2 hours. This multi-step process ensured effective infiltration and stabilization of the catalytic materials within the membrane structure.

Upon completing the heat treatment of the first layer (CeO_2), the membranes underwent an additional preparation step to ensure proper infiltration of the second

layer using the $CeTiO_x$ sol. First, the membranes were placed into the vacuum for 15 minutes at 0.2 bar. Then, a 2% PVA solution was slowly introduced into the tubes, ensuring gradual and even filling. The vacuum was maintained at 0.2 bar for an additional 10 minutes to allow the PVA solution to thoroughly infiltrate the membrane pores. Afterward, the membranes were removed from the vacuum unit and allowed to dry at room temperature for 15-20 minutes. Once the PVA application was complete for all membranes, the $CeTiO_x$ infiltration process began. The same vacuum infiltration procedure was followed for the $CeTiO_x$ sol infiltration and finally, membranes were heat treated.

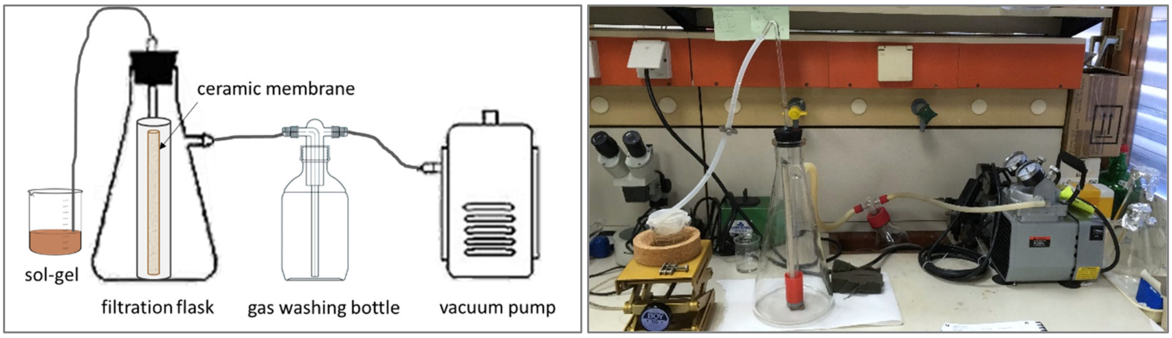

Figure S1. Scheme for the impregnation of the ceramic membranes with the vacuum impregnation technique.

Text S2. HPLC method for the qualification and quantification of the model compounds

For the quantification and qualification of the model compounds a method was developed in the HPLC-UV Agilent 1200 (Agilent Technologies, Santa Clara, CA, USA). The instrument was equipped with a quaternary pump, an autosampler, and an ultraviolet-visible detector. A C18 column (Microsorb-MV 100-5 250 × 4.6 mm) at a working temperature of 30 °C and flow 0.8 ml min^{-1} was used to separate the compounds. The method time was set to 40 minutes with an injection volume of 200 μL, and it can successfully separate and quantify the following compounds: carbamazepine, pCBA, diclofenac, and ibuprofen.

Three different solvents were used: (A) milli-Q water (H_2O), (B) 100% Acetonitrile (ACN), and (C) a solution containing 0.3% formic acid and 10% ACN. The selected OMPs were separated by changing the percentage of aqueous (H_2O) or organic (ACN) solvent while keeping the acidic water (pH 3) always at 10%. More hydrophilic compounds were analyzed with a higher H_2O:ACN mobile phase mixture, whereas more hydrophobic compounds with a lower H_2O:ACN ratio to avoid elution peaks

overlapping. The HPLC gradient of the mobile phase used is given below (Table S3): Carbamazepine, pCBA, diclofenac, and ibuprofen were detected at 284 nm, 236 nm, 274 nm, and 222 nm, respectively.

Table S3. *HPLC-UV mobile phase*

Time (min)	A (%)	B (%)	C (%)
0	80	10	10
4	80	10	10
5	80	10	10
8	40	50	10
9	40	50	10
27	40	50	10
28	0	70	30
36	0	70	30
38	80	10	10
40	80	10	10

Table S4. Parameters obtained from the Mercury Intrusion Porosimeter for the tested ceramic membranes.

		300 kDa MWCO			
**Parameters**	**Units**	**pristine**	**CeO_2**	**$CeTiO_x$**	**CeO_2+$CeTiO_x$**
Total intrusion volume at 206,6957 MPa	mL g^{-1}	0.077	0.020	0.078	0.070
Total pore area at 206,6957 MPa	m^2 g^{-1}	0.211	0.095	0.354	0.085
Median pore diameter (volume) at 0,3263 MPa and 0,039	mL g^{-1}	3897	3974	3924	3964
Median pore diameter (area) at 109,8838 MPa and 0,214	m^2 g^{-1}	62.37	3582	62.37	3745
Average pore diameter (4V/A)	nm	1468	3024	882.2	3264
Bulk density at 0,0033 MPa	g mL^{-1}	2.81	3.97	3.77	3.95
Apparent (skeletal) density at 206,6957 MPa	g mL^{-1}	3.59	5.56	5.33	5.44
Porosity	%	21.8	28.6	29.4	27.4

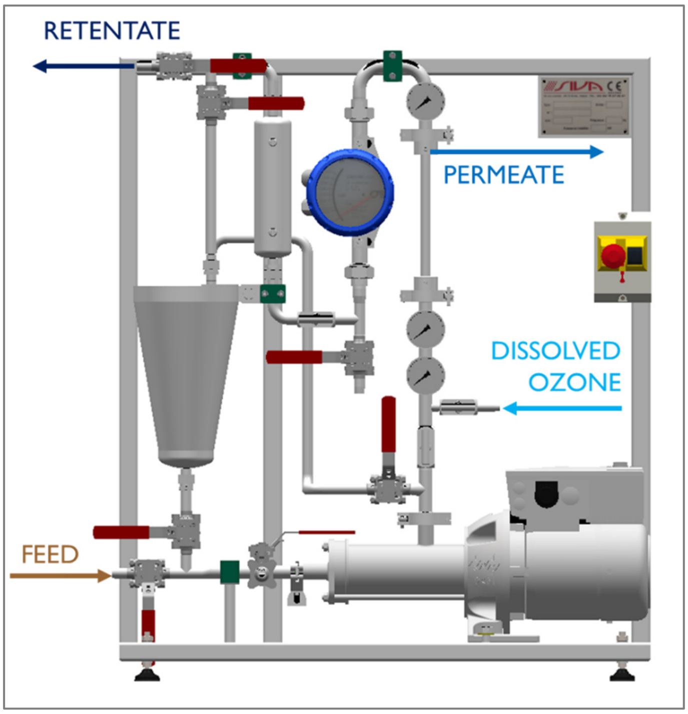

Figure S2. Custom-built crossflow filtration unit with an inside-out configuration (SIVA, France)

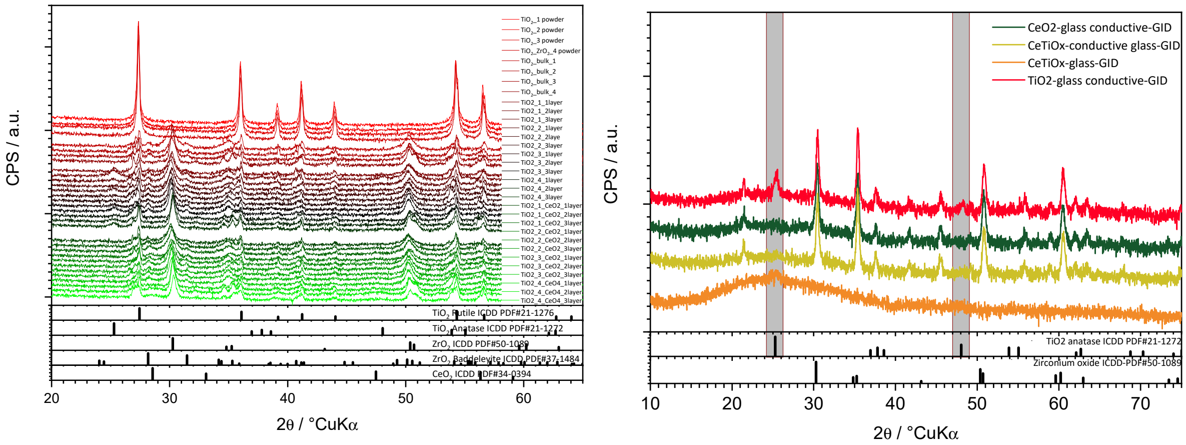

Figure S3. XRD analysis of the pulverized ceramic membranes before and after modifications (left) and XRD analysis of the deposited nanoparticles on a conductive glass to remove the noise of the background (i.e., ceramic membrane)

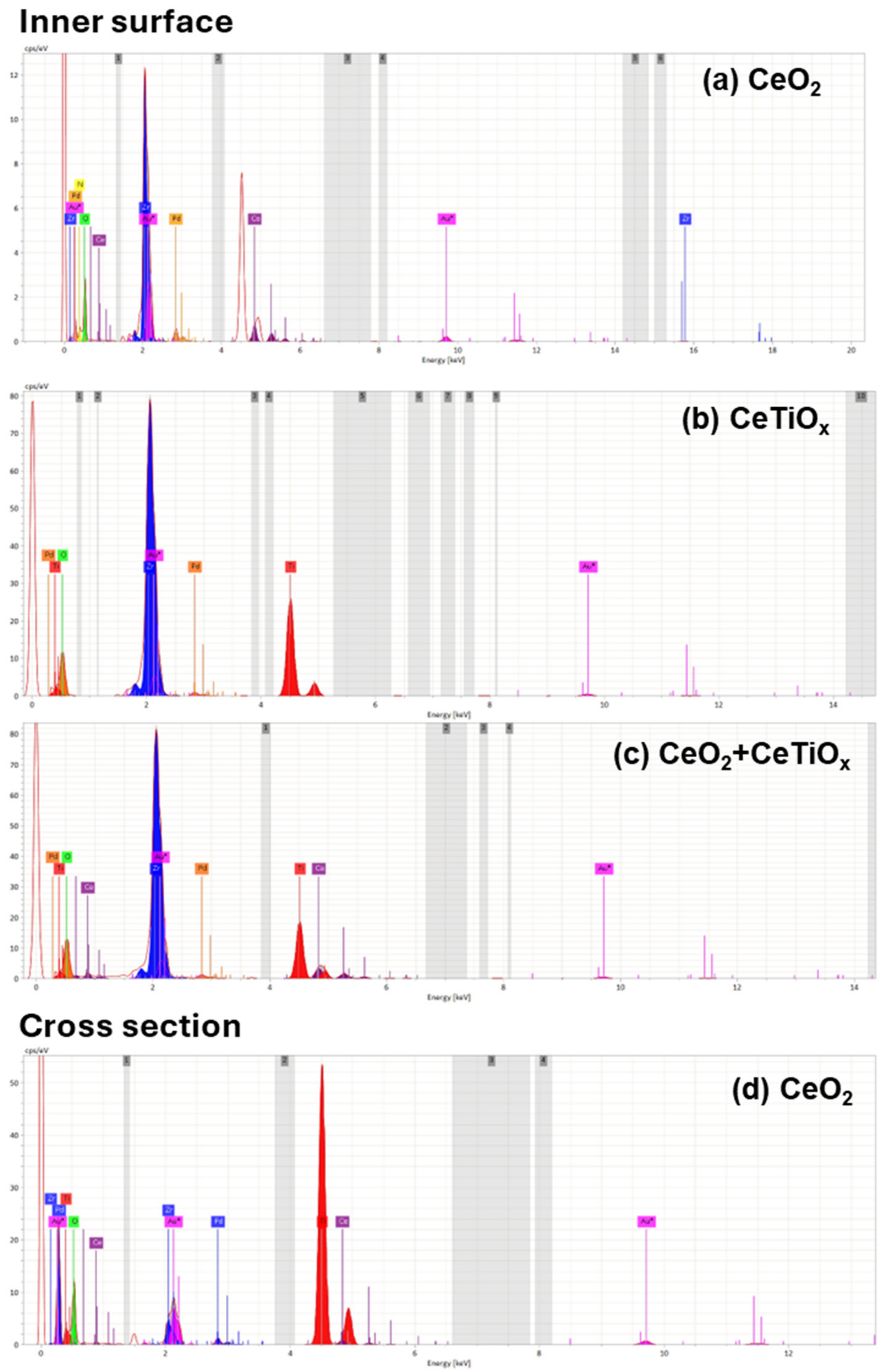

Figure S4. EDS spectra of the inner surface of the modified ceramic membranes for (**a**) CeO_2, (**b**) $CeTiO_x$, (**c**) CeO_2 + $CeTiO_x$, and the cross-section for (**d**) CeO_2.
